# *β-Defensin 2* Ameliorates Lung Injury Caused by *Pseudomonas* Infection and Regulates Proinflammatory and Anti-Inflammatory Cytokines in Rat

**DOI:** 10.3390/ijms150813372

**Published:** 2014-07-30

**Authors:** Zhenwei Shen, Lu Fang, Liming Zhao, Han Lei

**Affiliations:** 1 Department of Emergency Internal Medicine, Shanghai East Hospital, Shanghai 200120, China; E-Mail: zhweishen1@163.com; 2 Department of Nephrology, Shanghai East Hospital, Shanghai 200120, China; E-Mail: lufang052@163.com; 3 Department of Respiratory Medicine, Shanghai Changzheng Hospital, Shanghai 200003, China; E-Mail: lmzhao6@163.com; 4 Department of Respiratory Medicine, Shanghai East Hospital, Shanghai 200120, China

**Keywords:** *β-defensin 2*, lentiviral vector, shRNA, infection, cytokines, NF-κB

## Abstract

An important member of the defensin family, *β-defensin 2*, is believed to play an important role in defense against foreign pathogens. In the present study, we constructed lentiviral vectors to express and knockdown *β-defensin 2* in rat lungs. The results showed that the infection of *β-defensin 2* overexpression lentivirus and *β-defensin 2* shRNA effectively increased and suppressed the expression of *β-defensin 2* in rat lung, respectively. The overexpression of *β-defensin 2* mediated by the lentiviral vector protected lung from infection of *Pseudomonas aeruginosa*, but shRNA targeting *β-defensin 2* aggregated the damage of lung. In addition, we also found that *β-defensin 2* overexpression increased basal expression of anti-inflammatory cytokine such as IL-4, IL-10 and IL-13 and decreased levels of proinflammatory cytokines which include IL-1α, IL-1β, IL-5, IL-6, IL-8, IL-18, and TNF-α. Moreover, in the process of cytokine regulation, NF-κB pathway may be involved. Taken together, these data suggest that *β-defensin 2* has protective effects against infection of *Pseudomonas aeruginosa* in rat and plays a role in inflammatory regulation by adjusting cytokine levels.

## 1. Introduction

As an important member of the defensin family, *β-defensin 2* has extensive antibacterial activity, especially against Gram-negative bacteria and mold. It is mainly expressed in a variety of mucosa epithelial cells of the skin, bronchus and urogenital tract [1]. Its unique sterilizing mechanism is to destroy intact bacterial lipid membranes, so as to change the permeability of cellular membranes. Therefore, it is not easy to induce resistance in bacteria and can be used as a potential new antibiotic, which makes it a hot topic in anti-infection study [2]. Under normal circumstances, *β-defensin 2* is expressed rarely or at a low level, and is up-regulated to function in cells of various mucosa tissues exposed to stimuli, including proinflammatory cytokines and microorganisms. Different microorganism components can induce expression of different defensins [3,4]. In addition, as a signaling molecule, *β-defensin 2* can also induce and recruit inflammatory and immune cells, and influence cellular proliferation and differentiation [5].

Though *β-defensin 2* plays a crucial role in the body’s anti-infection and immune response, its effects in inflammatory response and the possible mechanism have not been elucidated clearly [6]. In the present study, the over-expression vector and RNA interference vector of rat *β-defensin 2* (*rBD2*) were packaged into lentiviral particles to infect rat lungs to up- or down-regulate *rBD2* level, pathologic changes of lung cells in inflammatory response were observed, and the expression levels and changes in relevant cytokines in lung tissues and peripheral blood were detected, to clarify the effects of *β-defensin 2* on these cytokines and to investigate the action of *β-defensin 2 in vivo*, so as to provide a theoretical basis for further research and application. 

## 2. Results

### 2.1. Construction and Analysis of cDNA and shRNA Expressing Vectors and Detection of Inhibitory Efficiencies

Segment was amplified from cDNA and inserted into the lentiviral vector and sequencing results showed that the *rBD-2* expressing vector was successfully constructed. Three short hairpin RNAs (shRNAs, shRNA1, shRNA2 and shRNA3) targeting different sites of the coding region of *rBD-2* were tested to select the most efficient interfering RNA sequence to inhibit rBD-2 expression. The co-transfection results in 293T cells showed that shRNA1 showed the highest efficiency in the three shRNA sequences, decreasing *rBD2* mRNA and protein by 82% and 63% (Data not shown). So, the lentiviral small hairpin RNA targeting *rBD2* (LV-shrBD1) was used for subsequent experiments.

**Figure 1:**
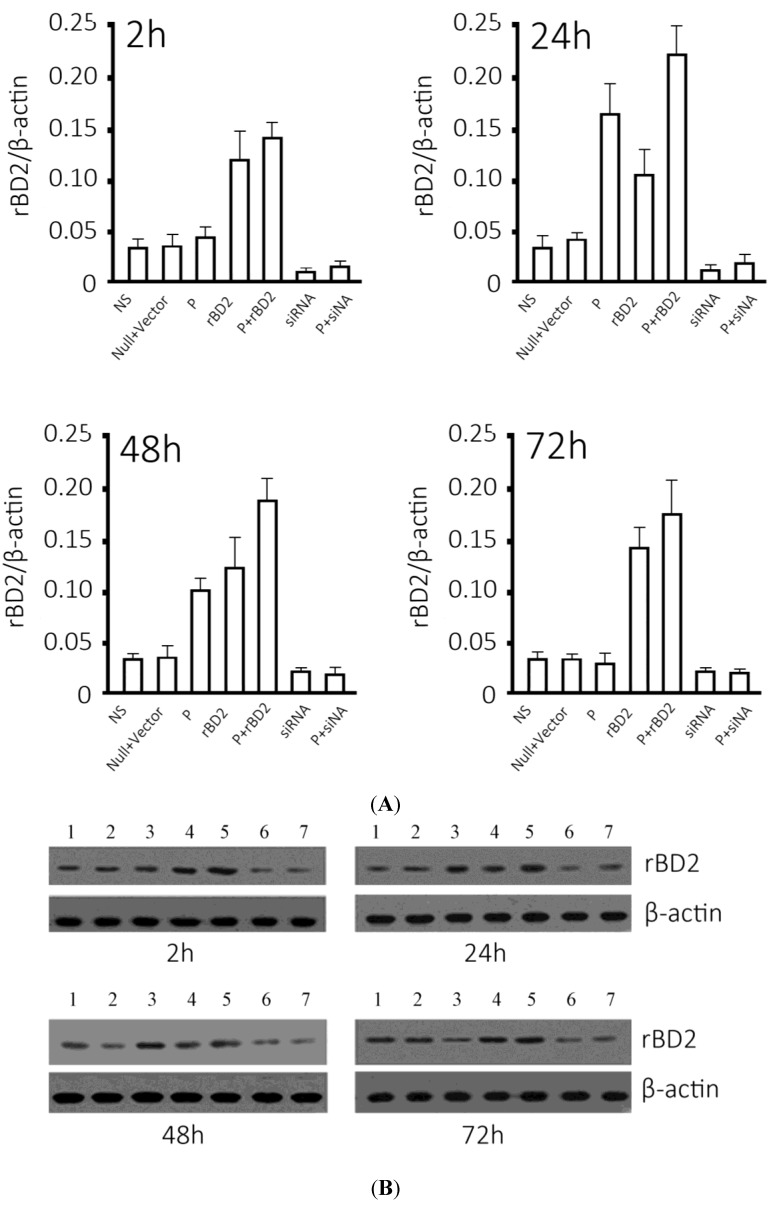
Overexpression and knockdown of *rBD2* mRNA and protein. (**A**) Relative *rBD2* mRNA levels normalized by *β-actin* at indicated time points. Data were expressed as mean ± SD. Groups: normal saline group (NS), group treated with null lentivirus (null vector), *Pseudomonas aeruginosa* infected group (P), group treated with *rBD2* expression lentivirus (*rBD2*), *Pseudomonas aeruginosa* infected group pretreated with *rBD2* expression lentivirus (P + *rBD2*), group pretreated with *rBD2* shRNA lentivirus (shRNA) and *Pseudomonas aeruginosa* infected group pretreated with *rBD2* shRNA lentivirus (P + shRNA); (**B**) Representative western blots of *rBD2* at indicated time points. *GAPDH* serves as an internal loading reference. Lanes: normal saline group (1); group treated with null lentivirus (2); *Pseudomonas aeruginosa* infected group (3); group treated with *rBD2* expression lentivirus (4); *Pseudomonas aeruginosa* infected group pretreated with *rBD2* expression lentivirus (5); group pretreated with *rBD2* shRNA lentivirus (6) and *Pseudomonas aeruginosa* infected group pretreated with *rBD2* shRNA lentivirus (7).

### 2.2. Effective Overexpression and Knockdown of rBD2 mRNA and Protein in Rat Lung Mediated by Lentiviral Particles

Lentiviral was successfully packaged for infection of rat lung tissues through trachea cannula. The *rBD2* mRNA in the normal saline group and the null vector groups varied little at different time points, so the expression levels were used as control. Infection of *Pseudomonas aeruginosa* substantially increased *rBD2* mRNA, and a peak was observed at 24 h and then decreased day by day. The injection of *rBD2* overexpression lentiviral particles increased *rBD2* mRNA in the groups uninfected and infected by *Pseudomonas aeruginosa*, and the latter showed the highest level of *rBD2*, which peaked at 24 h and fell gradually (Figure 1A). Lentiviral-mediated shRNA significantly suppressed *rBD2* mRNA expression in rat lungs. Though infection of *Pseudomonas* increased *rBD2* mRNA in *rBD2* knockdown group at 24 h, the level was obviously lower than that of control groups (Figure 1A).

Western blotting results showed a similar trend (Figure 1B): *Pseudomonas aeruginosa* increased *rBD2* protein in rat lungs peaking at 24 h, and infection of *rBD2* expression lentiviral particles enhanced the increase and the infection of shRNA expressing lentiviral particles suppressed the increase. 

### 2.3. Histological Changes in Rat Lungs of rBD2 Over-Expression and Knockdown

**Figure 2:**
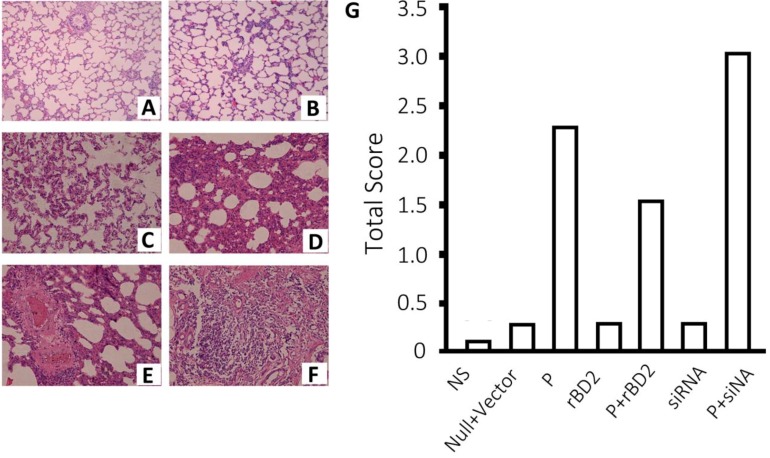
Histological images of lungs of rats subjected different treatment. Representative microscopic images of lungs in (**a**) group treated null lentivirus; (**B**) group treated with *rBD2* expression lentivirus; (**C**) group pretreated with *rBD2* shRNA lentivirus; (**D**) *Pseudomonas aeruginosa* infected Group; (**E**) *Pseudomonas aeruginosa* infected group pretreated with *rBD2* expression lentivirus and (**F**) *Pseudomonas aeruginosa* infected Group pretreated with *rBD2* shRNA lentivirus 24 h after injection of *Pseudomonas aeruginosa* when applied; (**G**) Average total pathology scores of the normal saline group (NS), group treated with null lentivirus, *Pseudomonas aeruginosa* infected group (P), group treated with *rBD2* expression lentivirus (*rBD2*), *Pseudomonas aeruginosa* infected group pretreated with *rBD2* expression lentivirus (P + *rBD2*), group pretreated with *rBD2* shRNA lentivirus (shRNA) and *Pseudomonas aeruginosa* infected group pretreated with *rBD2* shRNA lentivirus (P + shRNA).

Microcopy indicated that the lung tissues of the normal saline group, null vector group, *rBD2* group and shRNA group were normal with scores lower than 0.25 (Figure 2). The infection group showed significant histological changes including alveolar wall destruction, inflammatory cell infiltration of alveolar space and interstitium, with a score of 2.25 (Figure 2). The lentiviral-mediated expression of *rBD2* reduced the total score to 1.5, with the samples showing partial alveolar wall hemangiectasis, neutrophil granulocytes in alveolar space and interstitium, and slight desquamation of epithelial cells in alveolar space. To the contrary, shRNA aggravated the pathologic changes induced by *Pseudomonas aeruginosa*, increasing the score to 3 (Figure 2). 

### 2.4. Pseudomonas aeruginosa Infection Level

**Figure 3:**
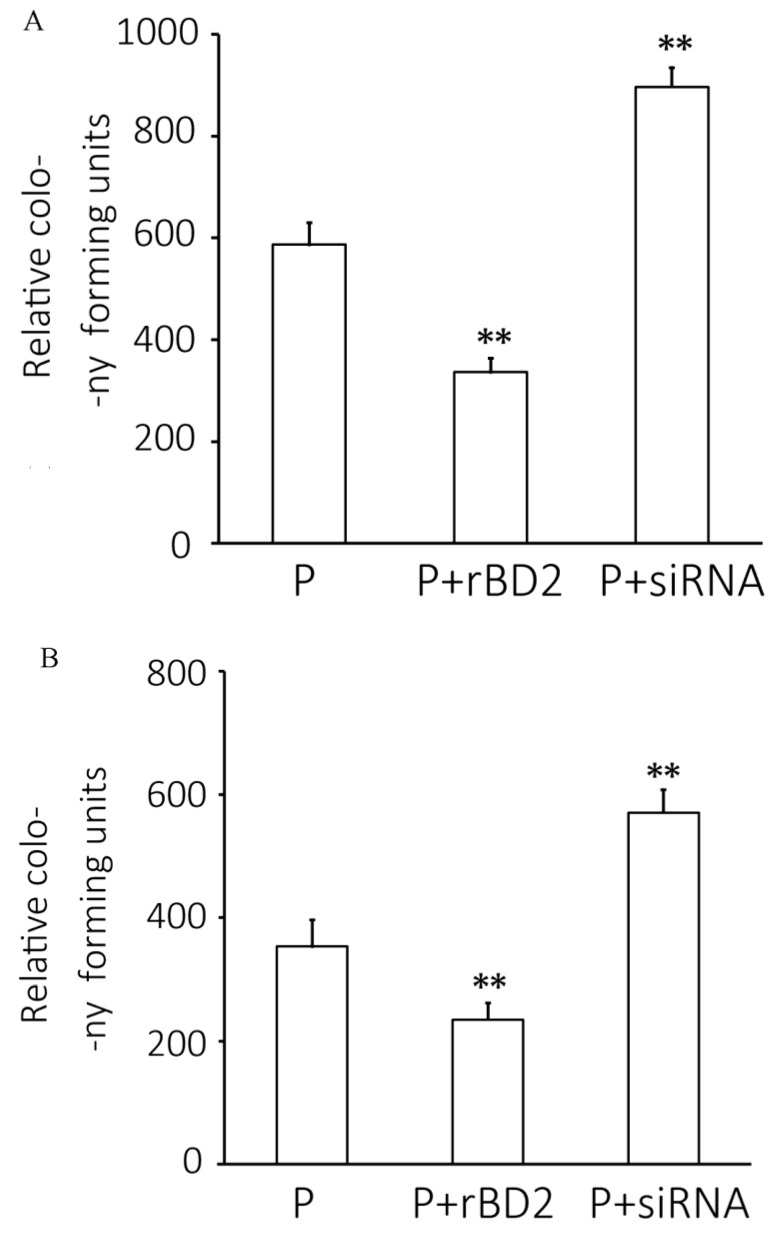
Counts of colony forming units (cfu) present in lung tissue homogenates (**A**) and bronchoalveolar lavage fluids (BALFs) (**B**). Data are expressed as expressed as mean ± SD (*n* = 8). P, *Pseudomonas aeruginosa* infected group; P + *rBD2*, *Pseudomonas aeruginosa* infected group pretreated with *rBD2* expression lentivirus (P + *rBD2*); P + shRNA, *Pseudomonas aeruginosa* infected group pretreated with *rBD2* shRNA lentivirus (P + shRNA). ** *p* < 0.01 *versus Pseudomonas aeruginosa* infected group.

The colony-count results to determine the infection levels in lung tissue homogenates and bronchoalveolar lavage fluids (BALFs) are shown in Figure 3. Relative to the results from those only received *Pseudomonas aeruginosa*, overexpression of *rBD2* decreased levels of infection in lung tissues and in BALFs, by 42.6% and 33.9%, respectively, indicating that *rBD2* increased the body clearance of *Pseudomonas aeruginosa*, whilst knockdown of *rBD2* increased *Pseudomonas aeruginosa* infection level in BALFs and lung tissue, by 52.8% and 60.6%, respectively. 

### 2.5. Effects of rBD2 on Cytokines

**Figure 4:**
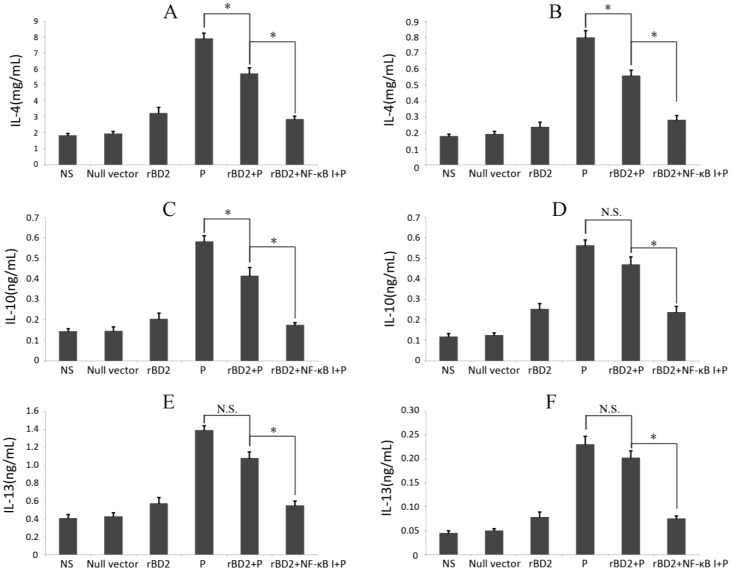
Effects of *rBD2* on anti-inflammatory cytokines. Quantitative analysis of IL-4 (**A**,**B**); IL-10 (**C**,**D**) and IL-13 (**E**,**F**) in lung tissue homogenates (**A**,**C**,**E**) and peripheral blood samples (**B**,**D**,**F**) in each group. Data were expressed as mean ± SD (*n* = 6). Groups: normal saline group (NS), group treated with null lentivirus (null vector), *Pseudomonas aeruginosa* infected group (P), group treated with *rBD2* expression lentivirus (*rBD2*), *Pseudomonas aeruginosa* infected group pretreated with *rBD2* expression lentivirus (*rBD2* + P), group pretreated with *rBD2* expression lentivirus and the inhibitor of NF-κB (*rBD2* + NF-κB I). * *p* < 0.05, and N.S. no significant difference.

**Figure 5:**
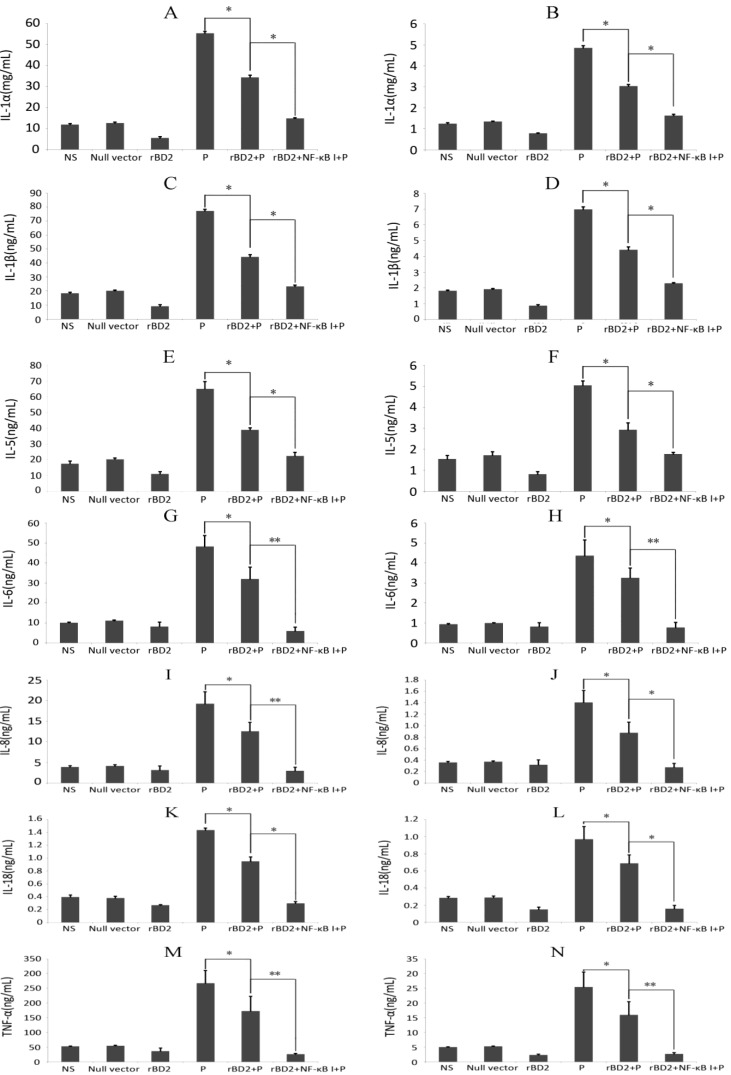
Effects of *rBD2* on proinflammatory cytokines. Quantitative analysis of IL-1α (**A**,**B**); IL-1β (**C**,**D**); IL-5 (**E**,**F**); IL-6 (**G**,**H**); IL-8 (**I**,**J**); IL-18 (**K**,**L**); and TNF-α (**M**,**N**) in lung tissue homogenates (**A**,**C**,**E**,**G**,**I**,**K**,**M**) and peripheral blood samples (**B**,**D**,**F**,**H**,**J**,**L**,**N**) in each group. Data were expressed as mean ± SD (*n* = 6). Groups: normal saline group (NS), group treated with null lentivirus (null vector), *Pseudomonas aeruginosa* infected group (P), group treated with *rBD2* expression lentivirus (*rBD2*), *Pseudomonas aeruginosa* infected group pretreated with *rBD2* expression lentivirus (*rBD2* + P), group pretreated with *rBD2* expression lentivirus and the inhibitor of NF-κB (*rBD2* + NF-κB I). * *p* < 0.05, and ** *p* < 0.01.

Since cytokines are closely related to infection and clearance of pathogens, we also evaluated the secretion of cytokines, the results showed that *rBD2* elevated the expression of anti-inflammatory cytokines, IL-4, IL-10 and IL-13 (Figure 4), and decreased the levels of proinflammatory cytokines, IL-1α, IL-1β, IL-5, IL-6, IL-8, IL-18, and TNF-α (Figure 5). The infection of *Pseudomonas aeruginosa* significantly increased the expression of all cytokines mentioned above. In the rats infected by *Pseudomonas aeruginosa*,over-expression of *rBD2* significantly attenuated the increases in nearly all detected cytokines induced by infection, notwithstanding whether it increased or decreased the basal expression levels of these cytokine in absent of infection (Figure 4 and Figure 5). In addition, the inhibitor of NF-κB significantly attenuated the effect of *rBD2* on nearly all the cytokines that we detected. 

## 3. Discussion

Studies on function and action mechanisms of defensins have increased gradually and become a hot anti-infection research area. However, most of those were carried out *in vitro*, so its major specific biological activities and roles and action mechanisms *in vivo* remain unclear, requiring further *in vivo* investigations. Since *rat BD2* is homologous to the human *BD2*-the conservative residues such as six cysteine and other lysine and arginine residues [7], we chose rat as a model to investigate the role *BD2* plays *in vivo*.

Lentiviral vectors are HIV-based replication-defective retroviral vectors [8], which can accommodate multiple promoters, infect both replicating and non-replicating cells at high efficiency [9,10,11,12,13]. In this study, we successfully constructed the recombinant vectors for *rBD2* expression and knockdown, and packaged respective viral particles. The infective lentivirus solution was introduced through the trachea cannula to rat lungs and RT-PCR and western blotting results showed that the lentiviral system efficiently delivered *rBD2* protein or blocked *rBD2* in lung tissues in a dose-dependent way; for overexpression, the optimal dose was 200 μg/100 g. In addition, the approach provided a local expression of protein or shRNA, avoiding side effects caused by overall expression or knockdown of *rBD2*. Moreover, due to gene integration, *rBD2* expression level was increased steadily since 12 h after infection and lasted to 72 h. In short, all these results suggested that the lentiviral system provided a stable and efficient way to adjust *rBD2* levels *in vivo* for further investigation. 

Inflammatory response can enhance expression of defensins, and in turn, defensins can stimulate dendritic cells and T cells through chemotaxis induction or directly, to affect the immune regulation and inflammation [14]. Human *BD2* has a strong chemotactic function, to recruit more neutrophils to clear pathogens, and to induce mast cells to aggregate, activate, and release histamine, by this way to participate in immediate hypersensitivity reactions [15,16]. Studies have shown that the inducible expression of *hBD2* gene may be associated with the development of acute lung injury (ALI), and its high concentration in lung secretion has protective effect on ALI [17]; In ARDS patients, defensing levels in BALF are highly correlated the level of IL-8, indicating that lung injury may be related to accumulation of neutrophils in the lung and the release of defensins [18]. Thus, the investigation on the change of *β-defensin 2* *in vivo* may contribute to elaboration of the details of occurrence and development of inflammatory response.

Histological examination showed that overexpression of *rBD2* reduced inflammation induced by *Pseudomonas aeruginosa* and increased the clearance of bacteria in the lung tissues and BALF, indicating that *rBD2* helps the body to resist *Pseudomonas aeruginosa*. Although some studies have showed that defensins have a dose-dependent cytotoxic effect, for instance the 24-h treatments with >50 µg/mL of *HBD-2* were cytotoxic to human bronchial epithelial cells (HBECs) [19], no significant cytotoxicity or inflammation was observed in the experiment. The reason may be that the concentration of *rBD2* was not enough to affect normal cells *in vivo*. High *rBD2* level helped to reduce pathologic change and tissue damage, and *rBD2* knockdown aggravated inflammatory response, coincident with the results reported by other literature [20,21]. The main reason maybe the anti-bacteria function reduced the damage caused by pathogen, and its regulation of inflammatory factors reduced inflammation. 

Cytokines regulate cellular immune interactions and are produced by lymphocytes, monocytes, macrophages, and, for some cytokines, also fibroblasts, neutrophils, endothelial cells, or mast cells [22]. Lung infection immunity initiated by *Pseudomonas aeruginosa* infection is a complex system which consists of innate, non-specific, adaptive defenses, various immunity-related cells are involved in the whole process. To clarify a full range of impact of *β-defensin 2* on the secretion of cytokines in the immune system, we selected typical anti-inflammatory cytokine such as IL-4, IL-10 and IL-13 and proinflammatory cytokines which include IL-1α, IL-1β, IL-5, IL-6, IL-8, IL-18, and TNF-α to analyze the relation between *β-defensin 2* and changes of cytokines levels [23]. 

We found that the increased overexpression of *rBD2* increased anti-inflammatory cytokines such as IL-4, IL-10 and IL-13 in lung tissues and serum, and decreased levels of pro-inflammatory factors such as TNFα, IL-1β, IL-5, IL-6, IL-8 and IL-18, but there was no statistical difference to the saline control group, suggesting *rBD2* overexpression had no significant effect on inflammatory cytokines in lung tissues of normal rats. *Pseudomonas* infection increased all inflammatory factors in the model rats, and overexpression of *rBD2* significantly inhibited the increase of inflammatory factors. The inflammatory factors decreased gradually along with attenuated inflammation, but the decrease of anti-inflammatory cytokines lagged behind pro-inflammatory factors, indicating that *rBD2* accelerated the weakening process of inflammation. 

Previous studies on interaction between cytokines and defensins have produced controversial results [19,24,25]. Sakamoto N *et al.* have found *HNP-1* but not *hBD-2* increase expression of IL-8 and IL-1α, suggesting that α and β defensins may have different effects on the synthesis of cytokines in airway epithelial cells [19]. Biragyn *et al.* proved that as an endogenous ligand for Toll-like receptor 4, murine *β-defensin-2* can induce strong TH1 adaptive immune responses *in vitro* [24]. Boniotto *et al.* reported that human *β-defensin* promotes IL-8 secretion in PBMC, and selectively induce secretion of IL-6 and IL-10, suggesting that defensins may play a role in connection of innate immunity and the acquired one [25]. 

NF-κB plays a vital role downstream of TLR in a variety of cellular activities and actively involved in the regulation of *hBD-2* expression [26,27,28]. After activation, it is relocated into the nucleus, regulating the transcription of inflammatory cytokines and cell proliferation (anti-apoptotic) genes to regulate inflammatory responses and lymphocyte maturation [29]. In this study, we also investigated whether NF-κB is involved in mechanism of action of *rBD2*. 

Compared to the effect of *rBD2* overexpression in *Pseudomonas aeruginosa* infected rats, the combination of the NF-κB inhibitor and *rBD2* overexpression in *Pseudomonas aeruginosa* infected rats exhibited a stronger inhibiting effect on inflammatory factors. For this point, more research is needed to assess whether the inhibition of NF-κB increased the anti-inflammation effects of *rBD2* or impaired the inflammation induced by *Pseudomonas aeruginosa* directly.

A possible reason may be that as an endogenous ligand of TLR4, *rBD* may activate NF-κB to trigger the expression of some cytokines, so as to activate T cells and induce T lymphocytes transform to the Th2 cells or recruit Th2 cells, which can be blocked by NF-κB inhibitor, but further studies are needed.

## 4. Experimental Section

### 4.1. Expressing Vectors Construction, Virus Package and Infection

Total RNA was extracted from rat epithelium cells, and reversely transcribed into cDNA using M-MLV reverse Transcriptase (Takara BIO, Otsu, Shiga, Japan), which was used to amplify *rBD2* coding sequence with a pair of primers: F: 5'-GGAATTCGCCACCATGAGGATCCATTACCTTC-3' and R: 5'-ATAAGAATGCGGCCGCCTACTTTTTCTTGCAGCA. The product was purified, recovered and then ligated to the linear lentivector (System Biosciences, Mountain View, CA, USA). The ligation mixture was transformed into the competent DH5α strain and the positive clones were selected, and the plasmid was extracted and then analyzed by PCR and sequencing. 

According to the sequence of *rBD2* (AF068861), three shRNA sequences targeting its coding region were designed, and three pairs of complementary DNA oligos were synthesized, annealed, and inserted into the linear lentivector. The resultant plasmids were selected and confirmed by sequencing. The RNAi vectors were co-transfected with the *rBD2* expression vector into 293 cells. 48 h later, the transfection efficiency was evaluated by fluorescent microscopy of enhanced green fluorescent protein expression, and total RNA and protein were extracted for determination of *rBD2* mRNA and protein, thus the most sufficient shRNA sequence was selected for subsequent experiments. The most efficient siRNA and shRNA sequence used in the article are as follows: siRNA: 5'-TGAGGATCCATTACCTTCT-3', shRNA: 5'-GATCCTGAGGATCCATTACCTTCTcttcctgtcagaAGAAGGTAATGGATCCTCATTTTTG-3'. Following the manufacturer’s instructions, the expressing vector or shRNA vector and Lentivirus Package plasmid mix were co-transfected into 293T producer cells using Lipofectamine™ 2000 (Invitrogen, Carlsbad, CA, USA). The supernatants were collected 48 h later and cleared by centrifugation and filtering through 0.45 µm PVDF membranes. Viral titer was evaluated by gradient dilution.

### 4.2. Animal Grouping and Treatment

Sprague-Dawley (SD) rats (male/female 1:1, 240 ± 10 g), provided by the Experimental Animal Center of the Second Military Medical University (Shanghai, China), were housed under controlled conditions. Procedures involving animals and their care were in accordance with the guidelines of the Experimental Animal Ethics Committee (No. 2010-1-1). Fifty six rats were randomly divided into seven groups with eight rats each: Group 1 was served as control (injected with normal saline, 250 μL/100 g body weight, twice daily for three days); Group 2 was injected with null lentivirus at 250 μL/100 g body weight, twice daily for three days; Group 3 received an infusion of live *Pseudomonas aeruginosa* (5 × 10^8^ CFU/mL at 300 μL), which was distributed to both lungs evenly; Group 4 and 5 were injected with 1 × 10^5^ ifu/μL *rBD2* expression lentiviral solution at 150 μL/100 g body weight, twice daily for three days, and Group 5 received an infusion of live *Pseudomonas aeruginosa* (5 × 10^8^ CFU/mL at 300 μL) 24 h after the last injection of *rBD2* expression lentiviral solution; Groups 6 and 7 received the injection of 1 × 10^5^ ifu/μL *rBD2* shRNA lentiviral solution at 150 μL/100 g body weight, twice daily for three days, and Group 7 were infused with live *Pseudomonas aeruginosa* (5 × 10^8^ CFU/mL at 300 μL) 24 h after the last injection of *rBD2* shRNA lentiviral solution. Rats were sacrificed 2, 24, 48, 72 h after infusion of bacteria or 26, 48, 72, 96 h after the last administration for the groups which were not subjected to bacteria. Lung tissue samples were collected, washed with normal saline and stored in liquid nitrogen for subsequent experiments. 

### 4.3. Determination of rBD2 mRNA in Rat Lungs by Quantitative RT PCR

Total RNA was isolated from the whole lung tissue using Trizol and reversely transcribed into cDNA using M-MLV Reverse Transcriptase (Takara BIO, Otsu, Shiga, Japan). The following primers were used for PCR amplification of *rBD-2*: F: 5'-GTCGCCCCTTTCAGCCTTTACT-3' and R: 5'-TTTTTCTTGCAGCATCTCACTCT-3'. PCR parameters were as follows: 40 cycles of denaturation at 95 °C for 10 s, annealing at 60 °C for 20 s and extension at 72 °C for 20 s. *β-actin* was used as a reference to normalized *rBD-2* levels using the 2^ΔΔ*C*t^ method. Each RNA sample was run in triplicate. The QRT-PCR primers and PCR parameters were as follows: Forward primer of: 5'-AGAGGGAAATCGTGCGTGAC-3' and Reverse primer: 5'-CCATACCCAGGAAGGAAGGCT-3'; 40 cycles of denaturation at 95 °C for 10 s, annealing at 60 °C for 20 s and extension at 72 °C for 20 s.

### 4.4. Western Blotting

Total protein samples were extracted from lung tissues and quantified using the bicinchoninic acid protein assay kit (Pierce, Rockford, IL, USA). Protein samples were separated by SDS-PAGE and transferred to PVDF membranes (Millipore, Billerica, MA, USA). The membranes were blocked in TBST containing 5% nonfat milk at room temperature for 2 h and incubated with the antibodies against *rBD-2* (SANTA CRUZ, Dallas, TX, USA) and GAPDH (SANTA CRUZ, Dallas, TX, USA) at 4 °C overnight. The bands of *r-BD2* and *β-actin* was detected with ECL chemiluminescence substrates (Pufei, Shanghai, China) using HRP-conjugated secondary antibody (SANTA CRUZ, Dallas, TX, USA).

### 4.5. Pathological Examination

The grouping and treatment were the same as described above, and the lung tissues were isolated from the rats sacrificed 24 h after injection of *Pseudomonas aeruginosa.* The fresh lung specimens were fixed in 4% PFA, embedded in paraffin, cut and baked, and subjected to hematoxylin and eosin (H&E) staining. The morphological changes of H&E-stained tissue were analyzed by microscopy and scored by summing up scores of interstitial oedema, alveolar oedema, inflammatory infiltration, alveolar haemorrhage, and change in pulmonary atelectasis (0 = normal; 1 = mild; 2 = moderate; and 3 = severe). 

### 4.6. Determination of Pseudomonas Infection Levels

Tissue samples from left lungs were homogenized, centrifuged and the supernatants were spread on plates and incubated overnight at 37 °C. Final bacterial counts were given in CFU/mL. BALF was prepared by carefully instilling 3 mL of sterile normal saline into the right lung for three time. The aggregated BALF was collected, spread on plates and subjected to incubation and bacterial counting. 

### 4.7. Determination of Cytokines in Lung Tissues and Peripheral Blood

The rats were grouped and treated as described above. Twenty four hours later, the rats were sacrificed and the lung tissues were extracted and homogenized, centrifuged and the supernatants were collected. The peripheral blood samples were also collected. All samples were detected using ELISA assay kits for IL-1α, IL-1β, IL-4, IL-5, IL-6, IL-8, IL-10, IL-13, IL-18, and TNF-α according to the instructions of kits (Invitrogen, Carlsbad, CA, USA). 

### 4.8. Statistics

All experiments were performed in triplicate. The data were expressed as mean ± SD. Student’s *t*-test was used to determine the statistical significance of the data obtained. Difference with *p* < 0.05 was considered statistically significant.

## 5. Conclusions

In this study, we elevated expression of *rBD2* in lung mediated by a lentiviral vector and explored the effects of *rBD2* on inflammatory response *in vivo* by observing lung inflammatory reaction, bacterial clearance, change in proinflammatory cytokines and possible signaling pathway. We proved that elevated *rBD2* can mitigate the pathological change in inflammation and increase bacterial removal; *rBD2* has an obvious regulatory action on inflammatory cytokines, namely suppressing proinflammatory cytokines and promoting anti-inflammatory cytokines, so as to regulate Th1/Th2 balance, to reduce inflammatory response in lung and in the whole body. NF-κB may be involved in the regulation of cytokines by *rBD2*. 

To sum up, this is the first time to demonstrate that lentiviral-mediated *rBD2* overexpression in rat lung efficiently suppressed pathological changes caused by infection of *Pseudomonas aeruginosa*, reduced inflammatory response and improved clearance of bacteria. Our study on the function of *rBD2* to resist infection of *Pseudomonas aeruginosa* may shed light on the understanding of modulation of inflammatory response.
